# Advanced Chemical Looping Materials for CO_2_ Utilization: A Review

**DOI:** 10.3390/ma11071187

**Published:** 2018-07-10

**Authors:** Jiawei Hu, Vladimir V. Galvita, Hilde Poelman, Guy B. Marin

**Affiliations:** Laboratory for Chemical Technology, Ghent University, Technologiepark 914, B-9052 Ghent, Belgium; Jiawei.Hu@UGent.be (J.H.); Hilde.Poelman@UGent.be (H.P.); Guy.Marin@UGent.be (G.B.M.)

**Keywords:** CH_4_ reforming, catalyst-assisted chemical looping, oxygen carrier, metal oxides, structured nanomaterials, core-shell, bifunctional materials

## Abstract

Combining chemical looping with a traditional fuel conversion process yields a promising technology for low-CO_2_-emission energy production. Bridged by the cyclic transformation of a looping material (CO_2_ carrier or oxygen carrier), a chemical looping process is divided into two spatially or temporally separated half-cycles. Firstly, the oxygen carrier material is reduced by fuel, producing power or chemicals. Then, the material is regenerated by an oxidizer. In chemical looping combustion, a separation-ready CO_2_ stream is produced, which significantly improves the CO_2_ capture efficiency. In chemical looping reforming, CO_2_ can be used as an oxidizer, resulting in a novel approach for efficient CO_2_ utilization through reduction to CO. Recently, the novel process of catalyst-assisted chemical looping was proposed, aiming at maximized CO_2_ utilization via the achievement of deep reduction of the oxygen carrier in the first half-cycle. It makes use of a bifunctional looping material that combines both catalytic function for efficient fuel conversion and oxygen storage function for redox cycling. For all of these chemical looping technologies, the choice of looping materials is crucial for their industrial application. Therefore, current research is focused on the development of a suitable looping material, which is required to have high redox activity and stability, and good economic and environmental performance. In this review, a series of commonly used metal oxide-based materials are firstly compared as looping material from an industrial-application perspective. The recent advances in the enhancement of the activity and stability of looping materials are discussed. The focus then proceeds to new findings in the development of the bifunctional looping materials employed in the emerging catalyst-assisted chemical looping technology. Among these, the design of core-shell structured Ni-Fe bifunctional nanomaterials shows great potential for catalyst-assisted chemical looping.

## 1. Introduction

To bolster the rapid economic growth of modern society, the entire world is facing increasing demands on its energy sources. Although the consumption of non-fossil fuels (such as renewable energy and nuclear power) is projected to grow faster than fossil fuels (coal, petroleum and natural gas), the latter are expected to account for almost 80% of global energy use through 2040 [1,2]. Therefore, the continued dependence on fossil fuels for energy supply in the foreseeable future is inevitable. However, fossil fuel-based energy production is widely considered to be a leading cause for greenhouse gas emissions such as CO_2_, which is the largest contributor towards the global warming effect [3,4,5,6,7,8]. With the adoption of the Paris Agreement at the 21st Conference of the Parties of the United Nations Framework Convention on Climate Change (UNFCCC) in December 2015, scientists from all over the world are now being challenged with a new goal: To limit the global temperature increase to 1.5 °C above pre-industrial levels [9].

Hence, there is an urgent need for strategies to reduce CO_2_ emissions in various related fields, especially industrial production and electricity generation, which contribute almost half of total emissions [10]. Carbon Capture and Storage (CCS) is a central strategy, as it not only reduces the associated greenhouse gas emissions in conformity with global targets, but also provides the opportunity to allow for a more sustainable use of fossil fuel energy [11]. The applications of the CCS scheme are reflected in two groups of technologies: One is the capture of CO_2_ from power plants, followed by compression, transportation and permanent storage [12]; another is the combination with strategies to improve energy efficiency for the separation of CO_2_, or the conversion of CO_2_ to value added chemicals [13,14,15]. The recent progress in CO_2_ capture and separation technologies with respect to the feasible processes, as well as new materials have been reported in some reviews [3,7,16]. In the past few decades, the large-scale capture of CO_2_ using amine absorbers became commercially available and was mainly applied in CO_2_ separation from flue gases [17]. This conventional amine scrubbing for CO_2_ capture however increases the energy requirements of a plant by 25–40% [18].

**Figure 1:**
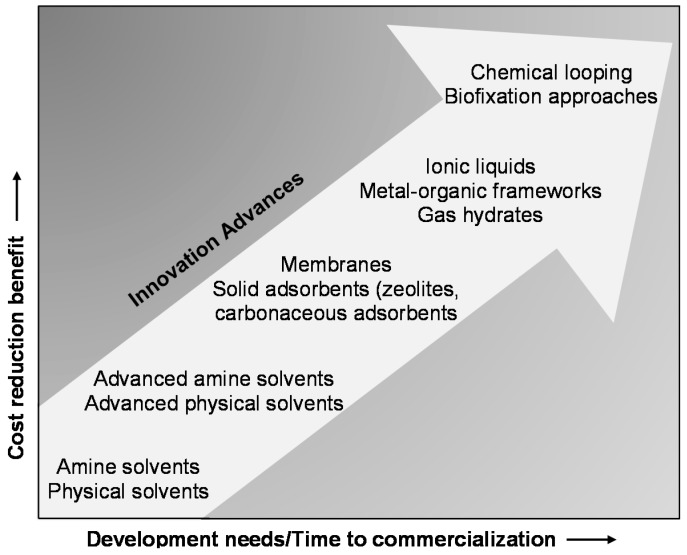
Innovation advances in CO_2_ capture technologies: Their cost reduction benefits vs. the remaining development needs/time to commercialization. Reproduced with permission from D’Alessandro, D.M. et al. [16], Angewandte Chemie International Edition; published by John Wiley and Sons, 2010.

From an economic point of view, there is need to improve current technologies for lowering the overall cost of CO_2_ capture and separation processes, which can be implemented from two aspects: Upgrading the efficiency of capture materials to decrease material expense, or designing novel process concepts to lower equipment cost. Figure 1 shows a series of important developments of CO_2_ capture technologies [16]. Evidently, more time and funding investments are required to commercialize the innovative techniques that possess higher cost reduction benefits. Among the innovating technologies, chemical looping technology has the potential to achieve inherent separation of CO_2_ from a fuel feed, thus eliminating energy intensive gas-gas separation costs, expectedly providing highest cost reduction benefits once it is fully commercialized. Furthermore, this technique can also provide an approach to convert CO_2_ into value added chemicals and energy sources (syngas, CO) [19,20,21,22,23].

**Figure 2:**
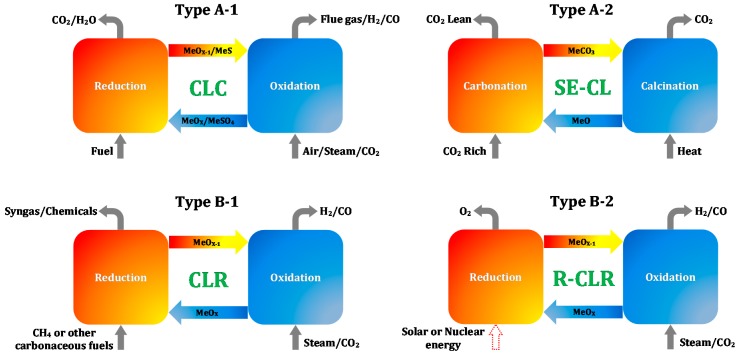
Chemical looping reactions involving CO_2_ generation (**top group A**) and no CO_2_ generation (**bottom group B**). Note: MeS: Metal sulfide; MeSO_4_: Metal sulfate; MeCO_3_: Metal carbonate; MeO: Metal oxide; MeO_X_ and MeO_X−1_: Oxidized and reduced form of metal oxide, respectively [21].

The concept chemical looping was first used by Ishida et al. [24] to describe a high-exergy efficiency combustion process that employs redox reactions of chemical intermediates. Later, this concept was extended to a broad range of applications, depending on the nature of a recyclable reaction scheme. Herein, a given reaction is decomposed into multiple sub-reactions, based on the reaction and regeneration of a solid mediator, called looping material (generally a metal oxide) performed in separate reactors or as subsequent steps in a single reactor [22]. Currently, the chemical looping processes can be categorized into two groups according to whether CO_2_ generation or separation is involved or not, as displayed in Figure 2 [21].

Extensive studies on group A have been reported [25]. The type A-1 process, also known as chemical looping combustion (CLC) [26], can be divided into two separate half-cycles: In the reduction half-cycle, the metal oxide is reduced, whilst the carbonaceous fuel is fully oxidized to CO_2_ and H_2_O; the reduced metal oxide is regenerated in the re-oxidation half-cycle by different re-oxidizing agents, such as air, steam or CO_2_, simultaneously producing power, H_2_ and CO, respectively. In addition, the gaseous O_2_ released from some metal oxides upon heating in the reduction half-cycle (i.e., oxygen uncoupling) can directly react with the fuel, which avoids the gas-solid reactions between the fuel and oxygen carrier. Based on this concept, an essential chemical looping process, i.e., chemical looping with oxygen uncoupling (CLOU), was established. Solid fuels, such as coal and biomass, can be used directly for combustion in CLOU process without gasification, showing advantage over a typical CLC process.

The type A-2 process uses a regenerable solid sorbent to achieve the in-situ capture of CO_2_ through a cyclic carbonation-calcination reaction before, during or after the carbonaceous fuel combustion or reforming [27]. It provides a viable CO_2_ control technology, i.e., sorption-enhanced chemical looping (SE-CL), for process intensification [15] or high-purity chemicals (CO_2_-lean) production [28]. The SE-CL process embodies two steps: CO_2_ is in-situ absorbed through the carbonation of a metal oxide, forming metal carbonate in the carbonator, afterwards the latter is calcined at higher temperature to regenerate the metal oxide, thereby releasing CO_2_.

In group B, a similar metal oxide regeneration process is involved as in group A, but the reactions in the former are not intended to produce CO_2_. The type B-1, chemical looping reforming (CLR), aims to reform carbonaceous fuels to syngas (a mixture of H_2_ and CO) or chemicals (e.g., ethylene) [29]. Utilization of CO_2_ can be achieved if it is used as an oxidizing agent in the re-oxidation half-cycle, turning it into chemical looping dry reforming (CLDR). Compared to conventional dry reforming (DR), application of CLDR brings two main advantages: First, since the looping material is regenerated in each cycle, deposited carbon can be removed by reacting with CO_2_. Second, as CO_2_ is used as an oxidizing agent for material regeneration in the re-oxidation half-cycle, this process can utilize more CO_2_ than conventional dry reforming; in case of CH_4_ reforming, the CO_2_ converted in CLDR is three times higher than in DR [23].

When using hydrocarbon fuels such as natural gas and biogas in CLDR, the kinetics of the reduction half-cycle is limited. The natural mixture of CH_4_ with CO_2_ (e.g., in biogas) significantly decreases the rate and degree of metal oxide reduction because CO_2_ also acts as an oxidizing agent. Both CO_2_ in the feed and as a product have this retarding effect on the metal oxide reduction, thus limiting the conversion of CO_2_ to CO in the re-oxidation half-cycle. In order to overcome these drawbacks, an extra catalyst component can be introduced to convert CH_4_ and CO_2_ into CO and H_2_, which then both reduce the metal oxide during the reduction half-cycle. This not only increases the CH_4_ and CO_2_ conversions, but also diminishes the oxidizing atmosphere because both CO and H_2_ are reducing agents. Based on this concept, catalyst-assisted chemical looping dry reforming (CCDR) was recently proposed as an intensified process with the goal of maximum utilization of two main greenhouse gases, CH_4_ and CO_2_, in terms of CO yield. This process is generally implemented over a bifunctional reactor bed, composed of either a physical mixture of a reforming catalyst and a metal oxide looping material, or a bifunctional looping material [15,23,30]. The entire CCDR process yields the dry reforming of CH_4_ into CO as an overall reaction, being a strongly endothermic process. Thermodynamic calculations indicate that an increase of temperature is necessary to obtain high CH_4_ and CO_2_ conversion, leading to larger energy requirements for heating the reactor, which highly increases the operating cost. Additionally, material deactivation due to active metal sintering will be aggravated under high-temperature conditions during dry reforming [31]. To counter these problems, CCDR was adjusted to yield a new process, called catalyst-assisted chemical looping auto-thermal dry reforming (CCAR) [20]. Here, a small amount of O_2_ is added to CH_4_ and CO_2_ to partially convert CH_4_ in an exothermic reaction (partial oxidation or combustion) [31,32,33,34,35], so that heat is generated in-situ, thereby compensating the strong endothermicity of the dry reforming reaction. Based on thermodynamic analysis, the whole CCAR process can run in thermoneutral conditions, i.e., ΔH = 0 kJ∙mol^−1^, with molar ratios of CH_4_:CO_2_:O_2_ = 1:2.4:0.3 at 600 °C or with molar ratios of CH_4_:CO_2_:O_2_ = 1:2:0.5 at 750 °C [20]. Owing to the inherent composition of biogas, typically containing CH_4_ (45~75%), CO_2_ (25~55%) and O_2_ (0.01~5%) [36], the CCAR can efficiently utilize biogas as fuel feed in the reduction half-cycle.

Compared to type B-1, the operation of type B-2 uses carbon neutral energy sources like solar and nuclear instead of carbonaceous fuels, providing a renewable chemical looping reforming (R-CLR) process. In R-CLR, considerable heat from solar or nuclear origin is imposed on the metal oxide to decompose it to metal and oxygen in the reducer. Subsequently, the metal is re-oxidized to metal oxide by steam or CO_2_, forming pure H_2_ or CO [37].

In light of the above overview of chemical looping processes, the key to success for chemical looping technology are the looping materials. Their adequacy in reactivity at temperatures below the melting point ensures the energy conversion efficiency during chemical looping. On the other hand, the contamination resistance and physical strength of a looping material at high redox reaction temperatures determine the durability of a chemical looping process. Further, the low price and environmental compatibility of the looping materials play a crucial role in the sustainability of the chemical looping technology. Therefore, major efforts have been made to develop the ideal chemical looping materials in the past decade. During the early stages of development, research focused on the metal oxide materials with a single function as CO_2_ accepter or oxygen storage, which are mainly used for conventional chemical looping combustion and reforming processes. The advances were reported in a few review papers [1,2,14,21,26,38,39]. More recently, a novel process, so-called catalyst-assisted chemical looping, was proposed with the aim of maximal CO_2_ utilization. Since then, the development of chemical looping materials has been directed towards the bifunctional material that integrates catalyst and oxygen storage material in one unit, achieving both catalytic fuel conversion and chemical looping CO_2_ utilization. In this review article, an update of the previous overview of chemical looping materials is given, together with an extension focusing on new insights in the development of bifunctional looping materials based on the latest studies.

## 2. Materials for Chemical Looping

Depending on the chemical looping process performed, the looping materials are mainly classified in two types: CO_2_ carrier and oxygen carrier, which are required to have high reactivity and sufficient stability, and to be cost-effective and environmentally friendly [40].

### 2.1. CO_2_ Carrier

Among all the solid CO_2_ sorbents, CaO is a proper material to act as CO_2_ carrier because of numerous advantages [41,42]: (1) low cost since it can be derived from limestone that is readily available; (2) non-toxic to the environment; (3) the spent materials can be further used in the cement industry after decarburization, supporting sustainable development in economy; (4) CaO may also serve as an alkaline catalyst in biomass conversion processes; and (5) the heat from the exothermic carbonation of CaO can be retrieved to compensate the high heat requirement during chemical looping. However, it is well known that the reactivity decay over cycles of CaO-based CO_2_ sorbents, particularly natural material-based sorbents, is inevitable. The main causes for the deactivation are particle sintering and attrition. Hence, many efforts have been made to improve their sintering resistance ability and mechanical strength, including doping of the material to suppress sintering, thermal or chemical pretreatment to achieve superior structural properties, development of new synthetic sorbents with good CO_2_ capture capacity and stability. Additionally, the development of feasible technologies for the reactivation of spent sorbents is of equal importance. The recent progress in CaO-based looping materials has been summarized by numerous review articles [43,44,45,46].

### 2.2. Oxygen Carrier

The nature of the oxygen carrier is a crucial matter, because a chemical looping process requires it to possess a certain redox activity and be able to withstand multiple cycles without large loss in the integrity of physical and chemical properties. Ideally, the oxygen carrier should function with reproducible kinetics at high temperature, and the materials that only show reactivity near or even above their melting point should be eliminated [1]. Oxygen transport capacity is another important property of an oxygen carrier. It is defined as the usable oxygen in the oxygen carrier during one redox cycle, which determines the fuel conversion and the circulation rate [2,47]. If chemical looping process is performed in a fluidized bed, the oxygen carriers additionally need to have sufficient mechanical strength to avoid attrition, which is quite common during their lifetime. In this sense, the attrition behavior of materials should be evaluated according to standard crushing tests under chemical looping operation conditions [39]. When carbonaceous and sulfur-containing fuels (fossil fuels and biomass) are used as feedstocks in chemical looping, issues like coke deposition, sulfur poisoning and high operation temperature inevitably result in deactivation of the oxygen carrier. Hence, properties like coke resistance, sulfur tolerance and thermal stability are of great importance for developing a proper oxygen carrier. In addition, the effect of ash formation on the performance of an oxygen carrier cannot be ignored, particularly in the utilization of biomass. Ash with low melting point alkali metals such as Na or K will promote agglomeration of the oxygen carrier [48,49], while high-acid ash (e.g., SiO_2_-rich ash) leads to the production of potassium silicates that facilitate particle sintering, suppressing the fuel conversion and CO_2_ capture efficiency [50]. Therefore, the resistance of an oxygen carrier to agglomeration is also a critical factor for its practical application. Eventually, the reactivity of oxygen carrier includes not only the theoretical conversion of fuel, relying on the thermodynamic properties of the materials, but also transport and reaction kinetics, which depend on composition, structure as well as texture properties like particle size, porosity and specific surface area [1].

**Figure 3:**
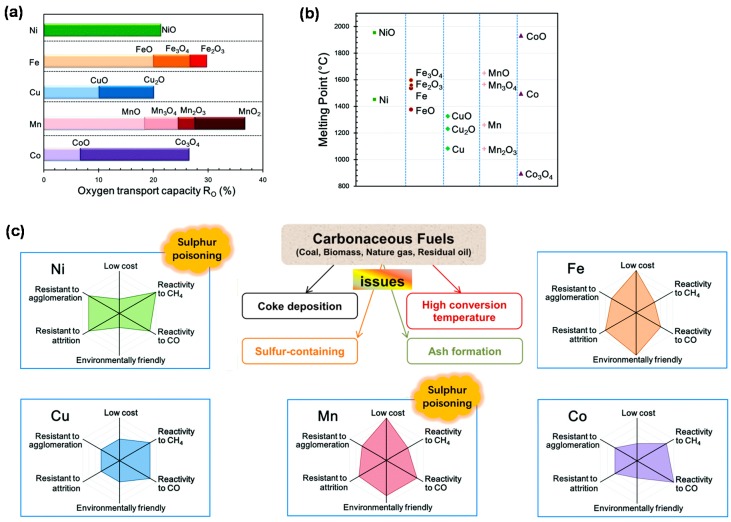
Comparison of Ni, Fe, Cu, Mn and Co-based oxygen carriers in term of oxygen transport capacity (**a**), melting point (**b**) as well as cost, reactivity, agglomeration or attrition resistance (**c**). The oxygen transport capacity R_o_ (%) is used to evaluate the maximum oxygen transport between the fully reduced (OC_r_) and oxidized (OC_o_) forms of the oxygen carrier (OC):R_o_ = (OC_o_ − OC_r_)/OC_o_. Adapted with permission from Zhao X. et al. [39], Energy & Environmental Science; published by The Royal Society of Chemistry, 2017.

Among various transition metals, Ni, Fe, Cu, Mn and Co-based materials are commonly considered to serve as oxygen carriers, although they present some disadvantages: Low reactivity and low oxygen transport capacity of Fe and Mn, low melting point and high agglomeration tendency of Cu, as well as high cost and negative health effects of Ni and Co. Figure 3 compares key information for these traditional oxygen carriers [39].

Despite of the toxicity and high cost, Ni-based material still receives major attention as a reliable reforming catalyst due to its high catalytic activity with CH_4_ and other light hydrocarbons [51]. Almost complete CH_4_ conversion can be obtained over Ni-based oxygen carriers during a CLR process. However, particle sintering and the loss of metallic Ni, owing to partial re-oxidation into NiO, along with the repetition of cycles cause a significant loss of catalytic performance [52]. In addition, Ni-based materials are sensitive to deactivation by sulfur poisoning and coke deposition. Improved Ni-based materials are necessary to be designed to tackle these drawbacks for further application.

Fe-based materials are commonly known as a good option for oxygen carriers due to their low price, high mechanical strength and environmentally friendly nature [39]. They almost have no tendency for carbon or sulfur deposition [53], making them promising candidates in all chemical looping processes involving carbonaceous and sulfur-containing fuels. Further, iron oxide presents the highest oxygen storage capacity from CO_2_ (0.7 mol CO_2_/mol Fe) over a broad range of operating temperatures (600~1800 °C) [22]. Thus, chemical looping dry reforming over Fe-based oxygen carriers has been one of the target technologies for CO_2_ utilization. However, within the multiple oxidized states of Fe system, only the transformation from Fe_2_O_3_ to Fe_3_O_4_ is suitable for CLC process due to thermodynamic limitations, leading to low oxygen transport capacity of Fe-based oxygen carriers. Other drawbacks also impede the application of Fe-based oxygen carriers, including relatively low reactivity towards gaseous fuels and the agglomeration related to magnetite formation [54]. The development of new-generation Fe-based oxygen carriers should focus on solving these issues.

The flexible redox behavior between the oxidized (CuO) and reduced (Cu or Cu_2_O) forms of Cu ensures that Cu-based materials have both high reactivity and oxygen transfer capacity. The advantages of Cu also include relatively low cost and toxicity, so Cu-based oxygen carriers have received greater attention from both industry and academia. Moreover, the negative influence of sulfur impurities on the performance of Cu-based oxygen carriers during a chemical looping process is not significant when the oxygen carrier-to-fuel ratio lies above 1.5 [53]. There are two main disadvantages that limit the application of Cu-based materials as oxygen carrier: One is attrition owing to their weak mechanical strength, another is agglomeration as a result of the low melting point of Cu. The former can be relieved through stabilizing Cu by inert promoters, such as Al_2_O_3_, ZrO_2_, TiO_2_ and SiO_2_ [55]. In order to solve the melting problem, keeping the operation temperature lower than 800 °C could be effective [39].

Similar to Fe, Mn has various oxidation states including MnO_2_, Mn_2_O_3_, Mn_3_O_4_ and MnO, among which Mn_3_O_4_ is the only oxide phase of Mn that exists at a temperature above 800 °C. Hence, only the redox transformation between Mn_3_O_4_ and MnO is applicable for the CLC process, showing a relatively low oxygen transport capacity. Additionally, Mn oxides were found to show low reactivity towards CH_4_. Similar to Ni-based materials, deactivation of Mn-based materials caused by sulfur contaminants cannot be ignored [56,57]. Nevertheless, Mn-based materials are still accepted to serve as oxygen carriers because of their low toxicity and cheap price. Some related research indicates that Mn promoted with typical materials like SiO_2_, Al_2_O_3_, TiO_2_, ZrO_2_ and MgAl_2_O_4_, can interact to irreversibly form highly unreactive phases [58,59], making them unsuitable as oxygen carriers. However, Mn-based materials using dopant-stabilized ZrO_2_ (applicable dopants such as MgO, CaO, Y_2_O_3_, La_2_O_3_ and CeO_2_ [60]) as support proved to possess not only high reactivity but also high structural stability and agglomeration resistance during redox cycles, which is prospective as promising Mn-based oxygen carriers [61].

Owing to the high reactivity and oxygen transport capacity, Co-based materials have been increasingly considered as good oxygen carriers, even though they also suffer from cost and environmental concerns. As the highest oxidation state of Co, Co_3_O_4_ is thermodynamically unstable above 900 °C and easily transformed into CoO. In this respect, the redox transformation between Co and CoO is commonly adopted in chemical looping processes [2]. However, CoO shows strong interaction with common promoter materials such as Al_2_O_3_, MgO and TiO_2_, forming unreactive phases like CoAl_2_O_4_, Mg_0.4_Co_0.6_O and CoTiO_3_, respectively. As a result, these oxygen carriers generally undergo fast deactivation during repeated cycles [62,63]. In contrast, the Co-based oxygen carrier with YSZ (yttria-stabilized zirconia) as support was found to exhibit good reactivity and high resistance to carbon formation in CLC process [62].

In summary, all of the above mentioned factors should be considered and balanced based on the involved feedstock and the desired chemical looping process when selecting an oxygen carrier. The total cost of the typical metal-based oxygen carriers follows the order: Co > Ni > Cu > Fe > Mn-based material (Figure 3), which includes the cost of active materials, promoter materials and the preparation section [2]. Overall, Fe- and Ni-based oxygen carriers are the most popular carriers for chemical looping, particularly for carbonaceous fuels-driven processes, because Fe oxides hardly suffer from carbon deposition and sulfur poisoning, while Ni shows excellent reactivity towards hydrocarbon fuels, comparable with the activity of noble metals (Pt, Pd and Rh).

## 3. Improvements to Oxygen Carriers

To meet the requirements of industrial chemical looping application, further improvements should be made to oxygen carriers to tackle their shortcomings. There are three main pathways: (1) the introduction of promoter and/or support; (2) the mixing of active metal oxides to integrate various advantages; and (3) the activation and stabilization of oxygen carriers by design of special structures. Here, we focus on reporting the advances related to improved Ni- and Fe-based oxygen carriers. The major improvement approaches, based on the promoters used, the material structure and the applied chemical looping processes, are summarized in Table 1.

### 3.1. Monometallic Materials

The promoter and support of monometallic materials can be classified as physically or chemically active based on their functions in oxygen carrier. The former usually serve as a physical barrier to improve thermal stability, while the latter contribute their redox properties to enhance reactivity [127].

Regarding to Ni-based oxygen carriers, series of heat-resistant oxides such as Al_2_O_3_ [64,65,66], SiO_2_ [58,59], TiO_2_ [67,68], ZrO_2_ and stabilized ZrO_2_ [69,70,71], as well as spinels like NiAl_2_O_4_ [72,73], MgAl_2_O_4_ and CaAl_2_O_4_ [74,75,76] are usually considered as adequate promoter materials. Among these, NiO/Al_2_O_3_ as an oxygen carrier demonstrates excellent reactivity, high thermal stability and strong resistance to carbon formation during chemical looping operation. However, the formation of an inactive NiAl_2_O_4_ spinel, owing to the strong interaction between NiO and Al_2_O_3_, leads to a partial loss of reactivity. As a result of NiAl_2_O_4_ formation, an excess of Ni should be added during material preparation to retain free NiO in the oxygen carrier. Accordingly, the NiO/NiAl_2_O_4_ oxygen carrier still shows high reactivity, although now a larger amount of Ni is required. Alternatively, the chemical passivation (i.e., the spinel formation behavior) can be used to enhance the inert function of Al_2_O_3_ promoter. Addition of MgO or CaO during the preparation of the NiO/Al_2_O_3_ oxygen carrier will form a more stable spinel like MgAl_2_O_4_ or CaAl_2_O_4_. The presence of such spinel in the oxygen carrier effectively suppresses the formation of NiAl_2_O_4_, maintaining the high reactivity of NiO during redox cycles and eliminating the need for more Ni. Generally, the use of other promoter materials shows problems with reactivity and mechanical strength: The use of TiO_2_ for Ni-based oxygen carrier was found to show slow reaction rate or even absence of reaction, because of the formation of the stable phase NiTiO_3_; the NiO/SiO_2_ oxygen carrier gives much lower reactivity, poor mechanical strength and deactivation with progress of cycling; the NiO/ZrO_2_ or NiO/stabilized ZrO_2_ oxygen carriers present good reactivity but their mechanical strength is weak.

**Figure 4:**
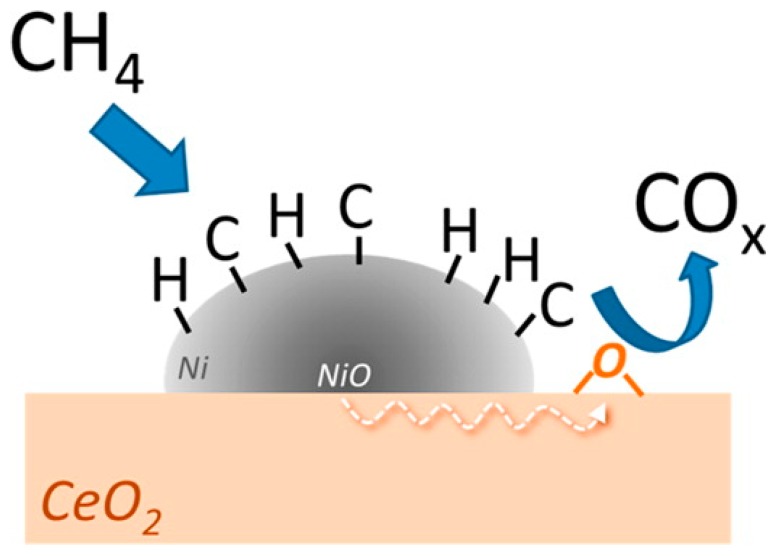
Schematic representation of a proposed mechanism for the synergistic effect between metal and reducible support in a NiO/CeO_2_ oxygen carrier for enhanced metal utilization in a chemical looping process. Obtained with permission from Bhavsar S. et al. [77]. Copyright (2013) American Chemical Society.

The use of a reducible oxide, particularly CeO_2_, as support for a Ni-based oxygen carrier, presents an obvious potential for enhanced redox properties of the oxygen carrier through a synergistic effect between Ni and CeO_2_ [77]. This synergistic effect may increase the utilization of Ni species due to the enhanced oxygen transfer from the CeO_2_ support to the Ni particles via the Ni-CeO_2_ interface, as shown in Figure 4: After the initial part of the reduction half-cycle, a Ni particle consists of two phases, i.e., metallic Ni (the outer layer of the particle) and NiO (the core of the particle). At the same time, CH_4_ is cracked on the surface of metallic Ni to form Ni-bound carbonaceous species, which afterwards can be gasified by lattice oxygen derived from nearby CeO_2_. The lost lattice oxygen in the support is refilled by oxygen from the core of the Ni particle (where the NiO phase is present), resulting in the continued reduction of the Ni particle.

Although Ni/CeO_2_ oxygen carrier shows high reactivity towards CH_4_, high carrier utilization and high resistance to carbon deposition in CLC process, a significant loss in surface area owing to sintering of CeO_2_ over repeated cycles indicates that the CeO_2_ support must be stabilized to make it applicable for industry. It has been confirmed that doping CeO_2_ with metals of different valency (such as La and Zr) can not only increase the thermal stability of CeO_2_ by maintaining the high surface area and preserving oxygen vacancies, but also further enhance the reducibility by increasing the oxygen mobility [128,129].

Regarding Fe-based oxygen carriers, promoter materials such as Al_2_O_3_ [78,79,80,81,82], MgO [83,130], TiO_2_ [84,85,86], SiO_2_ [87], MgAl_2_O_4_ [88,89,90,91,103], CaO [92,93], ZrO_2_ [94,95,96] and CeO_2_ [22,97,98,99,100] have received extensive scientific attention in the past decades. However, these promoters can easily lead to solid-solid transformation with iron oxides during a continuous cycling process [127]. Depending on the specific promoter, new phases like AlFe_2_O_4_, MgFe_2_O_4_, FeTiO_3_, and Fe_2_SiO_4_, Ca_2_Fe_2_O_5_ and CeFeO_3_ have a strong tendency to develop during the redox reactions. The so formed metal-ferrites can continue to serve as a physical barrier to prevent sintering of the iron oxide, but the overall oxygen carrier becomes less reactive in the presence of these phases, since the regeneration of the active iron oxides from the aforementioned metal-ferrites requires much stronger reducing and oxidizing conditions. As a result, the oxygen storage capacity decreases significantly [94,120]. Especially metal-ferrites such as Fe_2_SiO_4_ and Ca_2_Fe_2_O_5_ are unreactive and hard to decompose, and they usually cause a substantial decrease in the oxygen carrier reactivity and selectivity. Because the MgAl_2_O_4_ spinel forms more easily, the addition of an appropriate amount of Mg (the atomic ratio of Mg:Al = 1:2) to Fe_2_O_3_/Al_2_O_3_ could successfully alleviate the formation of FeAl_2_O_4_. Further, MgAl_2_O_4_ itself is also favored to act as an oxygen carrier support owing to its high thermal and chemical stability, as well as to its specific heat capacity. While using Fe_2_O_3_/MgAl_2_O_4_ as an oxygen carrier in a CLR process, an increase in activity and stability with the number of cycles was found. Marked by a strong inertness, the use of ZrO_2_ as a promoter has received a great deal of attention. An extensive study on the Fe_2_O_3_/ZrO_2_ oxygen carrier for chemical looping indicated that the physical structure of ZrO_2_ was relatively stable during cycling and that no new phase formed [94]. Additionally, it was observed that the use of Ca-, Mg- and Ce-stabilized ZrO_2_ as supports for Fe-based oxygen carriers yields a further enhancement of redox properties and thermal stability [54].

**Figure 5:**
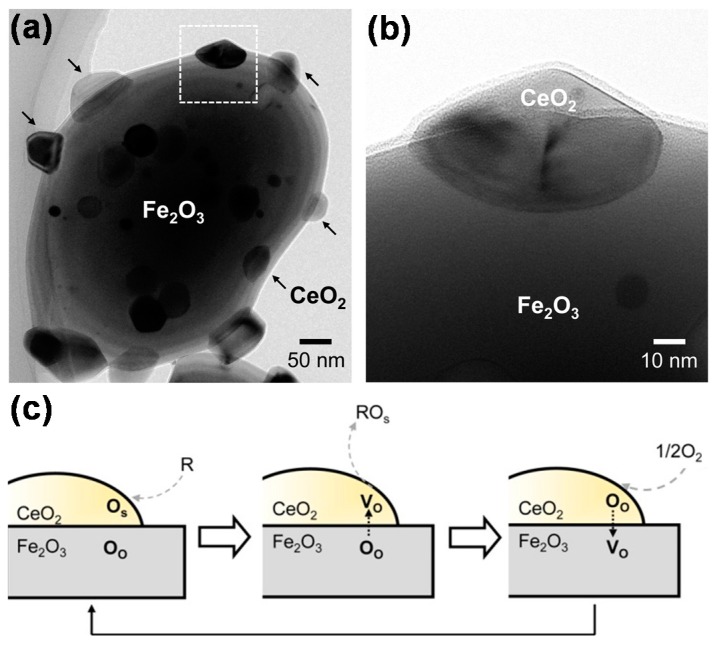
CeO_2_-grafted Fe_2_O_3_ oxygen carrier: Morphological structure: TEM image (**a**) and an enlargement of the square region (**b**); Schematic representation of its oxygen release and storage mechanism (**c**). V_O_: oxygen vacancy, O_O_: lattice oxygen, O_s_: surface oxygen. Adapted with permission from Machida M. et al. [98]. Copyright (2015) American Chemical Society.

CeO_2_ is an ideal candidate for promoting iron oxides in a chemical looping process, not only as a physical promoter, but also as a chemically active promoter due to its high activity towards methane or syngas oxidation by lattice oxygen, as well as its reasonable CO_2_ and H_2_O re-oxidation capacity [22,127]. The redox couple Ce^4+^ and Ce^3+^ facilitates oxygen storage and release from its bulk fluorite lattice. When the Ce^4+^ ions are replaced by lower valence cations (such as Fe^3+^), oxygen vacancies are created, which are considered to be active sites. After consuming the available surface oxygen for oxidation, extra oxygen can be transferred from bulk to surface more rapidly in a CeO_2_-Fe_2_O_3_ solid solution than in pure Fe_2_O_3_. The fast oxygen transfer from the bulk of Fe_2_O_3_ to its surface is enabled by the creation of oxygen vacancies owing to the CeO_2_ additive [1]. Therefore, the addition of CeO_2_ in Fe-based oxygen carriers enhances the reducibility of Fe_2_O_3_ by means of CeO_2_-Fe_2_O_3_ interaction. Machida et al. [98] proposed the concept that CeO_2_ and Fe_2_O_3_ can respectively serve as an oxygen gateway and an oxygen reservoir in a combined system, opening up a new type of Ce_2_O_3_-grafted Fe_2_O_3_ oxygen carrier that is different from the conventional Fe_2_O_3_/Ce_2_O_3_ solid solution materials. To validate this idea, they grafted CeO_2_ nanoparticles onto the surface of Fe_2_O_3_ particles by a nanometric colloidal sol technique (Figure 5a,b). A possible reaction mechanism for the oxygen release and storage over the CeO_2_-grafted Fe_2_O_3_ material (Figure 5c) was elaborated: Under reducing conditions, the surface oxygen of CeO_2_ (O_S_) is readily removed by a reducing agent (R), yielding an oxygen vacancy (V_O_). The latter is instantaneously re-filled by lattice oxygen (O_O_) supplied from the Fe_2_O_3_ bulk, actuating the reduction of Fe_2_O_3_. Subsequently, the reduced Fe species are quickly re-oxidized by dissociated O_2_ transferred from the CeO_2_ surface.

### 3.2. Mixed Oxide Materials

It is well accepted that complex metal oxides may provide better properties than the individual metal oxides. Therefore, oxygen carriers based on mixed oxides have been investigated by combining different active metal oxides in a single particle or mixing different oxygen carriers, each composed of a single metal oxide. The main objective is to combine the complementary advantages of different oxygen carriers for the practical application in a chemical looping process.

The CoO-NiO supported YSZ oxygen carrier was found to have high reactivity towards CH_4_, complete absence of coke formation and good regenerative ability in natural gas-fueled CLC, even though the formation of a NiCoO_2_ solid solution decreased the redox reaction rate to some extent [101]. Hossain et al. [102] reported that the addition of Co into a Ni/Al_2_O_3_ material not only enhanced the reducibility of the oxygen carrier due to the suppression of NiAl_2_O_4_ formation, but also inhibited the agglomeration of particles by maintaining consistent Ni dispersion throughout the redox cycles.

The incorporation of Fe into Cu-based oxygen carriers can relieve their agglomeration problem, owing to the high melting point of Fe_2_O_3_ [131]. Wang et al. [103] evaluated the performance of series of Fe_2_O_3_-CuO/MgAl_2_O_4_ oxygen carriers for coke-oven-gas-based CLC in a laboratory pressurized circulating fluidized bed system. The best working oxygen carrier was found to be Fe_2_O_3(45)_-CuO_(15)_/MgAl_2_O_4(40)_, which showed high reactivity and circulation stability. It was also found that the introduction of a NiO-based oxygen carrier into the Fe_2_O_3_/CuO-based oxygen carrier bed facilitated the reduction of the operating temperature in CLC process, due to the high reactivity of Ni towards CH_4_. Further, a layer-by-layer mixing mode of a NiO-based oxygen carrier and a Fe_2_O_3_/CuO-based oxygen carrier in a fixed-bed reactor avoids the formation of NiFe_2_O_4_ spinel during the reduction half-cycle, ensuring a good redox rate, thereby providing a promising reactor configuration candidate for an enhanced-CH_4_-reforming chemical looping process [132].

The bimetallic Fe-Mn-O system shows higher CH_4_ combustion (close to 100%) in the absence of oxygen or air, higher CO_2_ selectivity (almost no toxic CO byproduct) and more stable multicycle CLC performance, compared to the individual Fe or Mn oxides. Additionally, owing to the low cost of both Fe and Mn, the bimetallic Fe-Mn oxide can be considered as a very competitive oxygen carrier for the chemical looping process [104]. Moreover, both Fe-Mn-O and Ni-Mn-O systems have oxygen uncoupling properties, as a result, Fe_x_Mn_(1−x)_O and NiMn_2_O_4_ can be used as oxygen carriers for the CLOU process [105,106,107].

**Figure 6:**
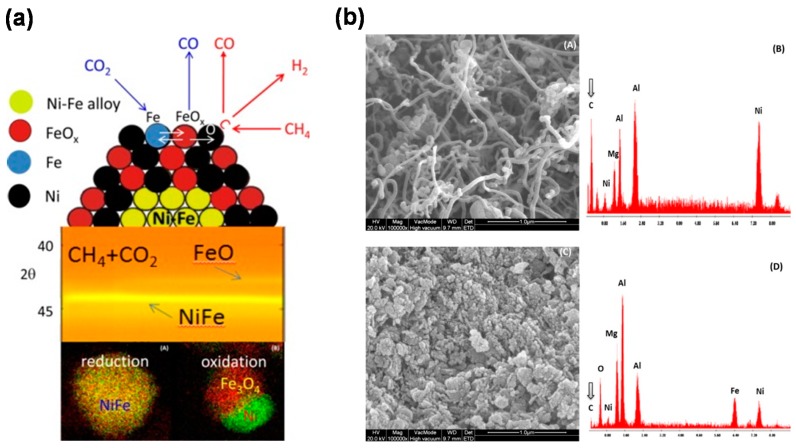
The bimetallic Fe-Ni/MgAl_2_O_4_ catalyst for enhanced carbon-resistant dry reforming: (**a**) The role of Fe for in-situ carbon removal from the catalyst surface during dry reforming; (**b**) Determination of the carbon resistance ability of the bimetallic catalyst by comparison of the spent samples of Ni/MgAl_2_O_4_ (A and B) and Fe_1.1_-Ni/MgAl_2_O_4_ (C and D) through SEM micrographs (left) and EDX analysis (right). Reproduced with permission from Theofanidis S.A. et al. [51]. Copyright (2015) American Chemical Society.

Continuing the comparison of Ni- and Fe-based oxygen carriers in Section 2.2: Ni shows best activity in CH_4_ conversion, but low resistance to carbon formation, whilst Fe has almost no trend to carbon deposition, with high oxygen storage capacity from CO_2_, but low reactivity towards CH_4_. Due to its low price and non-toxicity, Fe is advantageous from an economic and environmental point of view compared to Ni. Aiming to combine the complementary advantages of these two constituent metals, many efforts have been made to develop bimetallic Fe-Ni oxygen carriers via two different main strategies, physical [108,133] and chemical [23,108,109,110,111,112] mixing. Johansson et al. [133] reported that the addition of only 3 wt % NiO in the bed of a Fe-based oxygen carrier was sufficient to reach a very high CH_4_ conversion in CLC process. This is probably due to a synergy effect, i.e., Ni catalyzes the reforming of CH_4_ to CO and H_2_, which then both react with iron oxide at a considerably higher rate than CH_4_. In the case of chemically mixed oxygen carriers, in which the NiFe_2_O_4_ spinel usually forms, the CH_4_ conversion and reaction rate in CLC process follow the ranking: NiO > NiFe_2_O_4_> Fe_2_O_3_ [109], further indicating that alloying a less reactive metal—Fe—with a highly reactive one—Ni—can significantly improve the activity of the resulting bimetallic oxygen carrier. Compared to pure NiO and Fe_2_O_3_, NiFe_2_O_4_ is more suitable to serve as oxygen carrier for the CLR process, because the limited oxygen transfer capacity of NiFe_2_O_4_ avoids the complete oxidation of CH_4_, which results in an increased selectivity to syngas [112]. Alternatively, the addition of small amount of Fe into a Ni-based oxygen carrier or catalyst can obviously enhance the carbon resistance. Theofanidis et al. [51] prepared a series of bimetallic Fe-Ni/MgAl_2_O_4_ catalysts with different Fe/Ni molar ratios (from 0 to 1.5) to investigate the role of Fe on enhanced carbon resistance during CH_4_ dry reforming. They found that the process of dry reforming over bimetallic Fe-Ni catalyst follows the Mars—van Krevelen mechanism, where CO_2_ oxidizes Fe to iron oxide and CH_4_ is decomposed on Ni sites to H_2_ and surface carbon. The latter, simultaneously, can be oxidized by lattice oxygen from iron oxide, yielding CO (Figure 6a). During dry reforming, a Fe-Ni alloy is formed, which still constitutes an active phase and can be regenerated to metallic Ni and Fe_3_O_4_ by re-oxidizing by steam or CO_2_. Compared to monometallic Ni/MgAl_2_O_4_, the carbon deposition on the bimetallic Fe-Ni/MgAl_2_O_4_ is significantly suppressed (Figure 6b).

### 3.3. Structured Materials

#### 3.3.1. Perovskite Structure

Oxygen carriers with perovskite structure are receiving increasing attention owing to their enhanced redox properties, high oxygen mobility and thermal stability. Perovskite-structured oxide materials are typically formulated as ABO_3_, where A is a large alkaline earth or rare earth element and B is a smaller transition metal cation [134]. Where the A-site cation is responsible for the thermal resistance, the B-site cation will be the contributor to the reactivity, e.g., the catalytic activity in chemical looping reforming of CH_4_. As a result, the selection of B-site cations is crucial in the design of promising perovskite-structured oxygen carriers. In addition, the interaction between the A and B cations can affect the reactivity and selectivity during fuels conversion as well as the stability of the structure [1].

As well-established transition metal for oxygen carriers, Fe^3+^ has certainly been considered as a B-site cation. The performance of AFeO_3_ (A = La, Nd and Eu) was first investigated for selective oxidation of CH_4_ by Dai et al. [135]. It was found that, among the three AFeO_3_ candidates, LaFeO_3_ showed the best performance with respect to syngas production and stability during the redox cycling between CH_4_ and air at 900 °C. Because of the kinetic limitations, the reduction of LaFeO_3_ by CH_4_ is basically performed from Fe^3+^ to Fe^2+^, while deeper reduction is very difficult to achieve [113]. Further, partial substitution of the A-site cation by a low oxidation-state metal such as Sr leads to the formation of oxygen vacancies and a fraction of B-site cations in a higher valence state. A highly variable oxygen non-stoichiometry in the perovskite structure is hence introduced that can readily provide lattice oxygen, while subsequently the removed oxygen species can be replenished by gaseous oxygen. Substitution of La^3+^ by Sr^2+^ could cause electronic imbalance, but it can be compensated by the transformation of a fraction of Fe^3+^ to Fe^4+^. Therefore, La_1−x_Sr_x_FeO_3_ (x = 0 to ~1) perovskite oxides have been widely investigated as oxygen carriers in CLR processes [114,115,136]. Li et al. [136] proposed that there are two kinds of oxygen species on such perovskite oxides: One is the weakly-bound oxygen species which is active in complete oxidation of CH_4_ to CO_2_ and H_2_O, another is strongly-bound oxygen which is responsible for partial oxidation of CH_4_ to CO and H_2_. The activity of such perovskite materials was found to show a decrease when x increased above 0.5, probably owing to the suppression of CH_4_ decomposition by the Sr substitution [115]. The best performance was presented by La_0.7_Sr_0.3_FeO_3_, and during the reduction half-cycle of chemical looping steam reforming a maximum H_2_ yield was obtained over a simple mechanical mixture of La_0.7_Sr_0.3_FeO_3_ with an additional 5 wt % NiO [114]. On the other hand, the partial substitution of active B-site cations by aliovalent transition metal cations can lead to the enhancement of catalytic activity due to synergistic valence changes and introduction of non-stoichiometry–related microstructural defects into the lattice. Lori et al. [116] modified the “base case” material, La_0.7_Sr_0.3_FeO_3_, by transition metals such as Ni, Co, Cu and Cr. All the resulting perovskite-structured materials, with the general formula La_0.7_Sr_0.3_M_y_Fe_1−y_O_3_ (M is the additional transition metal), show the capability to act as suitable oxygen carriers in CLR process. The modification with partial substitution of Fe by Cr shows the best improvement compared to others. Additionally, La_0.7_Sr_0.3_Cr_0.1_Fe_0.9_O_3_ was found to have the optimum composition, showing highest CH_4_ conversion and syngas production, zero carbon deposition as well as good redox properties.

The application of CH_4_-based CLR over the perovskite-structured oxygen carriers, that provide lattice oxygen for CH_4_ conversion, represents an alternative in terms of high selectivity to syngas and elimination of the air separation process. However, the oxygen storage capacity of perovskite-structured metal oxides is relatively low compared to traditional metal oxides, and the oxygen transport capacity of the perovskites is less due to its slow reduction rate. Both disadvantages limit the application of the perovskite-structured oxide materials as industrial oxygen carriers.

#### 3.3.2. Core-Shell Structure

Fast redox kinetics and hence high oxygen transport rates of oxygen carriers are considered to be obstructed by the solid-state diffusion limitation due to the formation of a dense oxide overlayer during oxidation [137]. The transition from a kinetically controlled regime to a diffusion limited regime occurs when the overlayer reaches a critical thickness. Hence, an oxygen carrier with smaller particle size (i.e., nano-sized oxygen carrier) is expected to suffer less from such diffusion limitations by shifting the transition point towards close to complete oxygen carrier conversion, because, from a geometrical point of view, the fraction of the total particle volume constituted by a layer with constant thickness strongly increases with decreasing particle diameter [14]. Moreover, owing to the relatively slower oxidation kinetics with CO_2_ compared to air or steam, the use of nano-sized metal particles is expected to maximize the reactivity when CO_2_ is used as oxidizing agent [120]. In addition, it is believed that carbon deposition is not energetically favored over small metal particles, especially for Ni. As a consequence, the use of nano-sized Ni as oxygen carrier is very beneficial to chemical looping dry reforming of CH_4_ [138]. However, the fast deactivation of nanoparticles, due to severe sintering during high-temperature processes like chemical looping, poses a big issue for practical application. To counter this, oxygen carriers with a core-shell structure are designed, allowing stabilization of nano-size active metals by a thin and porous encapsulating shell (i.e., metal_core_@oxide_shell_ nanomaterial). This approach provides a promising strategy, not only to address the stability requirement, but also to improve the performance in terms of reactivity, redox properties and coke resistance, depending on the employed shell materials. Such core-shell structured nanomaterials have shown excellent performance in methane reforming [117,118,138,139,140] and chemical looping processes [14,40,119,120,121,122,123,124,141].

**Figure 7:**
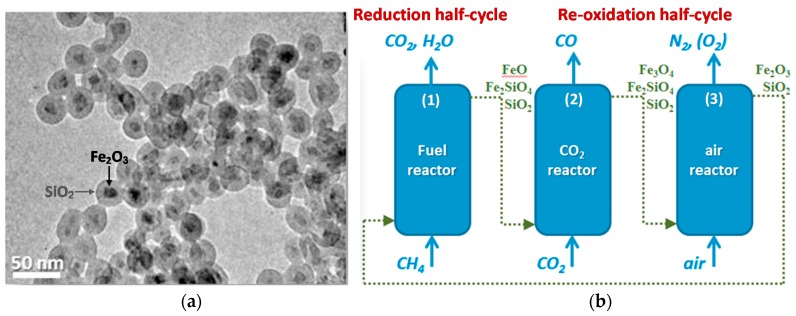
TEM image of a core-shell structured Fe_2_O_3_@SiO_2_ oxygen carrier (**a**) and schematic representation of a three-stage chemical looping dry reforming process for CO_2_ utilization (**b**). Reproduced with permission from Bhavsar S. et al. [14], Catalysis Today; published by Elsevier, 2014.

The first-generation metal_core_@oxide_shell_ nanomaterial of particular interest is a metal@SiO_2_ core-shell material, because of its easy preparation and low cost, providing great potential to be developed as commercial material, as well as the easy adjustment of its structural properties, such as porosity, specific surface area and shell thickness through modification of the synthesis method of the SiO_2_ shell [117,119]. Ni@SiO_2_ core-shell materials have received broad interest for CH_4_ reforming, as they turned out to be good redox catalysts or oxygen carriers, owing to their high activity and stability as well as their potential to resist coke deposition. Fe_2_O_3_@SiO_2_ core-shell materials (Figure 7a) however, show poor utilization of the oxygen carrier due to the formation of Fe_2_SiO_4_ during the reduction half-cycle. The active Fe cannot easily be restored from this Fe_2_SiO_4_ phase in the CO_2_ re-oxidation half-cycle, resulting in deactivation of material along with the proceeding cycles. To counter this deactivation, an additional air oxidation step is required to fully liberate active Fe from the Fe-silicate (Figure 7b), which leads to an increasing cost with process complexity [14,120].

**Figure 8:**
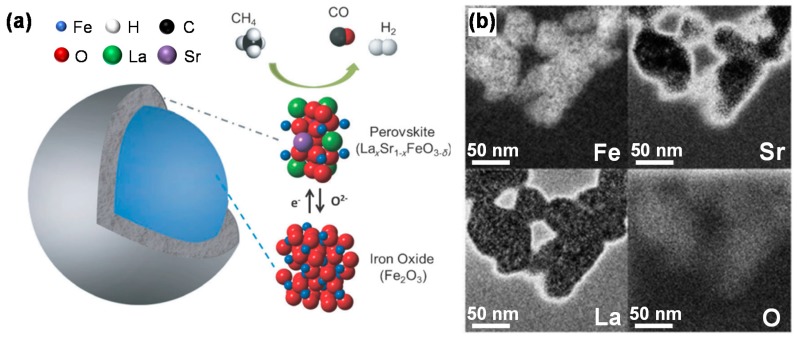
Schematics of the core-shell Fe_2_O_3_@La_x_Sr_1−x_FeO_3−δ_ material (**a**) and the EFTEM mappings of individual Fe, Sr, La and O elements (**b**), the brighter areas represent regions enriched with the element of interest. La and Sr are shown concentrated on the particle surface, while Fe is homogeneously distributed throughout, suggesting the formation of a core-shell structure. Reproduced with permission from Shafiefarhood A. et al. [121], ChemCatChem; published by John Wiley and Sons, 2014.

Although the above three-stage chemical looping dry reforming process allows sufficient utilization of the oxygen carrier via regaining Fe_2_O_3_ from the Fe_2_SiO_4_ phase in the air reactor, the low CO_2_ conversion in the CO_2_ reactor still limits the process efficiency in terms of CO_2_ utilization. Therefore, there is need to further explore reproducible core-shell structures. Li’s group designed a core–shell Fe_2_O_3_@La_x_Sr_1−x_FeO_3−δ_ material (Fe_2_O_3_@LSF), composed of a nanoscale Fe_2_O_3_ core (~50 nm) and a perovskite-structured La_x_Sr_1−x_FeO_3−δ_ shell (thickness of ~10 nm), which was successfully synthesized by using a sequential sol-gel method [121]. The performance of such material was investigated in CH_4_ partial oxidation: In this well-defined structure (Figure 8), syngas production from CH_4_ is enhanced by the highly selective LSF shell; the activity is improved with nano-size Fe_2_O_3_ which can provide more readily accessible lattice oxygen; the coating of Fe_2_O_3_ with a stable perovskite-structured shell does not hinder the recyclability of the material, but rather prevents Fe_2_O_3_ from sintering and carbon deposition, while improving the oxygen ion and electron fluxes. Thus, the proposed Fe_2_O_3_@LSF core-shell material was suggested to be a promising oxygen carrier for CLR processes. Later on, this group investigated the effect of core and shell compositions of MeO_x_@LSF (Me = Mn, Co and Fe) materials on the performance for CLR [122]. They found that the presence of Sr in the shell is favorable to stabilize Fe in the core-shell structure (i.e., prevents irreversible Fe leaching to the surface) by forming strontium hexaferrite (SrFe_12_O_19_) or a Sr deficient perovskite. However, these phases are more difficult to reduce, leading to a decrease of the overall reduction rate of the Fe_2_O_3_@LSF material. As a result, its redox activity is weakened.

**Figure 9:**
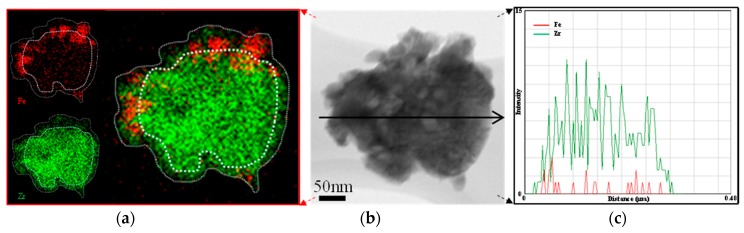
Morphology of Fe_2_O_3_/ZrO_2_@ZrO_2_ as core-shell nanomaterial: EDX element mappings of Zr and Fe (**a**), STEM image (**b**), and EDX line-scan (**c**). Contour lines of core and shell (dotted white line) in the mapping serve as guide to the eye. The black arrow in the STEM image represents the scanning route through the particle.

More suitable shell materials with chemical stability should be considered in order to maintain the long-term activity of core-shell structured Fe-based oxygen carriers. ZrO_2_ is of great interest as shell material due to its strong thermal and chemical stability. Li et al. [142] proposed a Ni@ZrO_2_ nanocomposite, constructed from nano-Ni particles encapsulated in a nano-ZrO_2_ framework, showing a strong ability to resistant sintering and coke deposition under reforming conditions. ZrO_2_ exists in different phases, among which the t-ZrO_2_ phase is dominant. It easily forms in nanoparticles and ultrathin films [143], where it contributes to a high specific surface area and oxygen conductivity. In a similar way, a core-shell material Fe_2_O_3_@ZrO_2_, with a thin, porous ZrO_2_ shell surrounding a nano-sized Fe_2_O_3_ core, can exhibit high reactivity and stability in chemical looping processes. The Fe_2_O_3_@ZrO_2_ material can be synthesized via nanocoating of Fe_2_O_3_ with ZrO_2_ through hydrolysis with a ZrO_2_ precursor [144,145]. In this process, two challenges need to be addressed: (1) the high tendency of nanoparticles to aggregate during the coating process, resulting in multiple cores within one shell [144]; (2) generally, the as-prepared Fe_2_O_3_ particles are non-densified, providing an unstable support for the shell, which easily collapses upon high-temperature reaction. To counter these drawbacks, a novel core-shell structured nanomaterial was proposed, Fe_2_O_3_/ZrO_2_@ZrO_2_ [40]. The core consists of Fe_2_O_3_ nanoparticles decorating a stable ZrO_2_ and it is coated afterwards with a porous thin ZrO_2_ layer (Figure 9)_._

**Figure 10:**
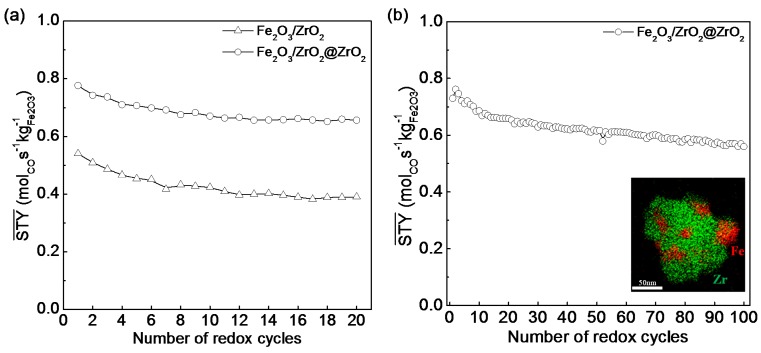
Time-averaged space-time yield ($\bar{\mathrm{STY}}$) of CO vs. number of redox cycles: (**a**) Comparison of CO $\bar{\mathrm{STY}}$ for non-coated Fe_2_O_3_/ZrO_2_ material and core-shell structured Fe_2_O_3_/ZrO_2_@ZrO_2_ nanomaterial during 20 redox cycles at 650 °C; (**b**) Stability of CO $\bar{\mathrm{STY}}$ over Fe_2_O_3_/ZrO_2_@ZrO_2_ during 100 prolonged redox cycles at 650 °C, the insert figure is the EDX element mapping of the spent core-shell material. Each cycle (10 min) is composed of 2 min H_2_ (5% in Ar) reduction, 2 min CO_2_ (pure) re-oxidation and 3 min intermediate He purging. Gas flow rates: 0.04 mmol∙s^−1^.

Applying Fe_2_O_3_ nanoparticles supported on stable crystalline ZrO_2_ as core material is advantageous; the Fe_2_O_3_/ZrO_2_ core avoids nanoparticle aggregation during the coating process, while at the same time providing a dense, stable support for the shell. The core material is covered with a ZrO_2_ shell by means of a general nanocoating process [146,147], using P-123 (a nonionic amphiphilic surfactant) as an active template layer on the core surface, upon which the shell is formed. Eventually, the template layer is calcined to decompose, leaving a mesoporous ZrO_2_ shell, permeable to reduction and oxidation gases to reach the iron oxide [148]. Compared to non-coated material, the core-shell Fe_2_O_3_/ZrO_2_@ZrO_2_ nanomaterial, designed to possess a stable Fe_2_O_3_/ZrO_2_ core and a mesoporous t-ZrO_2_ shell, exhibited excellent redox activity (higher CO_2_ conversion to CO at 650 °C) and stability (stable CO production, thermal-stable structure and high capacity to resist particle sintering) during 100 cycles of chemical looping conversion of CO_2_ (Figure 10).

## 4. Bifunctional Materials for Catalyst-Assisted Chemical Looping

When using Fe-based oxygen carrier as looping material, the reduction rate of iron oxide by hydrocarbon fuel is very slow, especially in the presence of CO_2_ (originating from feed and product). As a result, the conversion of fuel and CO_2_ is limited. Through addition of a catalyst component (commonly Ni), a bifunctional material, possessing an extra catalytic function for decomposition of hydrocarbon fuel into syngas (the latter ensures the formation of metallic Fe for adequate CO_2_ cyclic conversion), brings chemical looping towards a new stage—catalyst-assisted chemical looping dry (or auto-thermal dry) reforming (CCDR or CCAR), two processes which are designed for efficient conversion of hydrocarbon fuels accompanied by maximum CO_2_ utilization.

**Figure 11:**
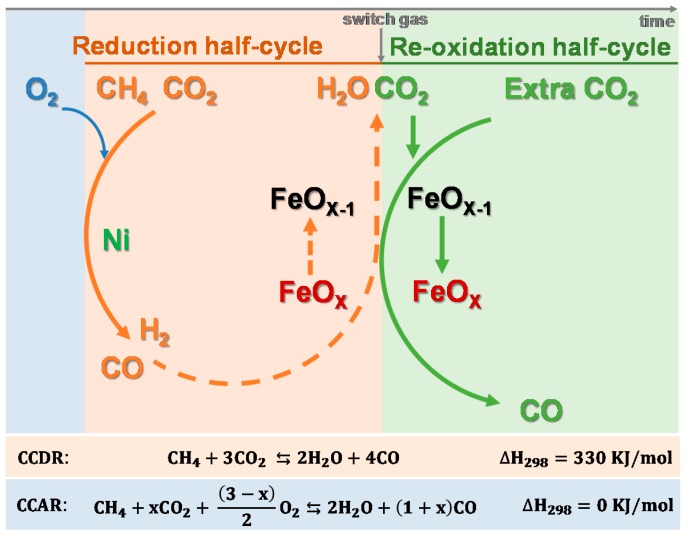
Schematic representations of catalyst-assisted chemical looping processes. Ni represents the Ni catalyst. FeO_X−1_ and FeO_X_ are the reduced and oxidized state of the iron oxide oxygen carrier, respectively. The equations in the bottom are the global reactions of the corresponding processes.

Figure 11 sketches the CH_4_-based catalyst-assisted chemical looping processes over a Ni-Fe bifunctional material. In the reduction half-cycle, the Ni catalyst converts CH_4_ and CO_2_ in syngas (a mixture of H_2_ and CO), which reduces the iron oxide oxygen carrier. Reduction of iron oxide by H_2_ and CO is significantly faster and deeper than by CH_4_ [23,149], which emphasizes the importance of the Ni catalyzed CH_4_ reforming process. Upon re-oxidation, the oxygen carrier is regenerated by feeding extra CO_2_, thereby producing CO. In such processes, the Ni-Fe bifunctional material is required to provide not only an optimum activity towards conversion of CH_4_ and CO_2_ into CO and H_2_, but also provide redox properties for interaction with both reducing gases (CO and H_2_) and oxidizing gases (CO_2_ and H_2_O). Therefore, the material must show functions of both reforming catalyst and oxygen carrier, i.e., exhibit a bifunctional character.

**Figure 12:**
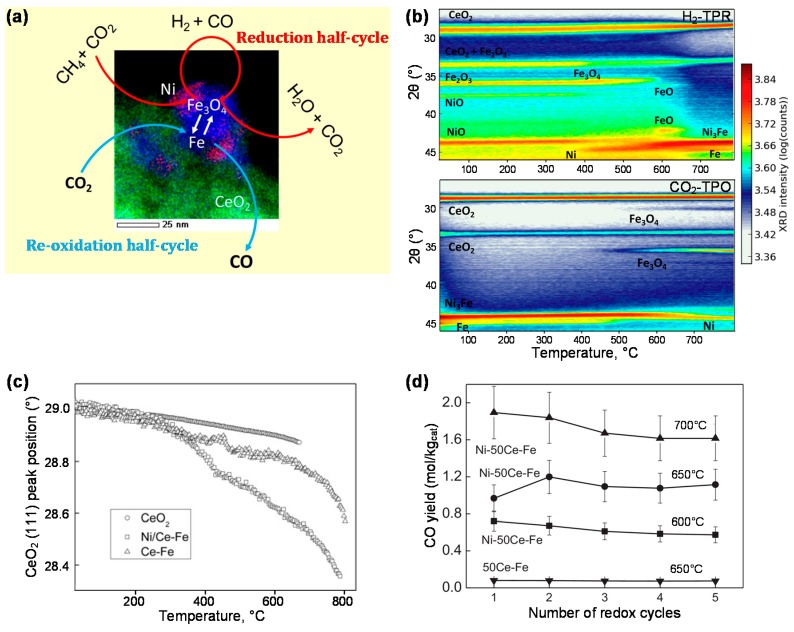
Catalyst-assisted chemical looping dry reforming over a 5 wt % Ni/CeO_2_-Fe_2_O_3_ (1:1) bifunctional material: (**a**) Schematic representation of the reactions in the two separated half-cycles, the STEM-EDX element mappings of Ni (red), Fe (blue) and Ce (green) show the morphological structure of the bifunctional material; (**b**) The evolution of crystal structure of the bifunctional material during in-situ H_2_-TPR and CO_2_-TPO. Measurement conditions include: temperature ramp rate: 20 °C/min, reducing gas for TPR: 5% H_2_/He, oxidizing gas for TPO: 100% CO_2_; (**c**) Variation of peak position of CeO_2_ (111) at 2θ = 29° during TPR for pure CeO_2_ (○), CeO_2_-Fe_2_O_3_ (∆) and Ni/CeO_2_-Fe_2_O_3_ (□). Same measurement conditions; (**d**) Comparison of CO yield during 5 redox cycles over CeO_2_-Fe_2_O_3_ oxygen carrier and Ni/CeO_2_-Fe_2_O_3_ bifunctional material at different temperatures. The error bar indicates twice the standard deviation. Adapted with permission from Galvita V.V. et al. [23], Applied Catalysis B: Environmental; published by Elsevier, 2015.

In previous work [23], a bifunctional 5 wt % Ni/CeO_2_-Fe_2_O_3_ material was prepared by impregnation of the required amount of Ni on a 50 wt % CeO_2_-Fe_2_O_3_ oxygen carrier. The working principle of the bifunctional material in CCDR is shown in Figure 12a. During the reduction half-cycle, the mixture feed of CH_4_ + CO_2_ is converted on Ni sites to CO and H_2_, which in turn reduce the oxygen carrier component (from Fe_3_O_4_ to metallic Fe), yielding CO_2_ and H_2_O. Subsequently, the reduced oxygen carrier is re-oxidized (from Fe to Fe_3_O_4_) by extra CO_2_, producing CO in the re-oxidation half-cycle. During H_2_-TPR of the Ni/CeO_2_-Fe_2_O_3_ bifunctional material, Fe_2_O_3_ is reduced to Fe_3_O_4_, and then FeO, finally transforming into metallic Fe. At around 350 °C metallic Ni appears due to the reduction of NiO. Interaction between Ni and Fe leads to Ni-Fe alloy formation from 580 °C onwards. This alloy remains stable up to 600 °C during CO_2_-TPO but is completely decomposed into Ni and Fe_3_O_4_ at 700 °C (Figure 12b). The addition of Ni to CeO_2_-Fe_2_O_3_ shows great promise towards generation of CO from CO_2_ in CCDR. As seen in Figure 12c, the position of the CeO_2_ (111) peak shows a downward shift indicating partial reduction. This shift is quite significant in Ni/CeO_2_-Fe_2_O_3_, while it remains limited in CeO_2_-Fe_2_O_3_ and doesn’t occur at these temperatures in pure CeO_2_, meaning the partial reduction is enhanced by the addition of Ni, leading to deeper reduction of the oxygen carrier. As a result, a higher CO yield can be obtained during the reduction half-cycle when feeding CO_2_. The CO yield over the Ni/CeO_2_-Fe_2_O_3_ bifunctional material is proven to be 10 times higher than over the CeO_2_-Fe_2_O_3_ oxygen carrier (Figure 12d).

**Figure 13:**
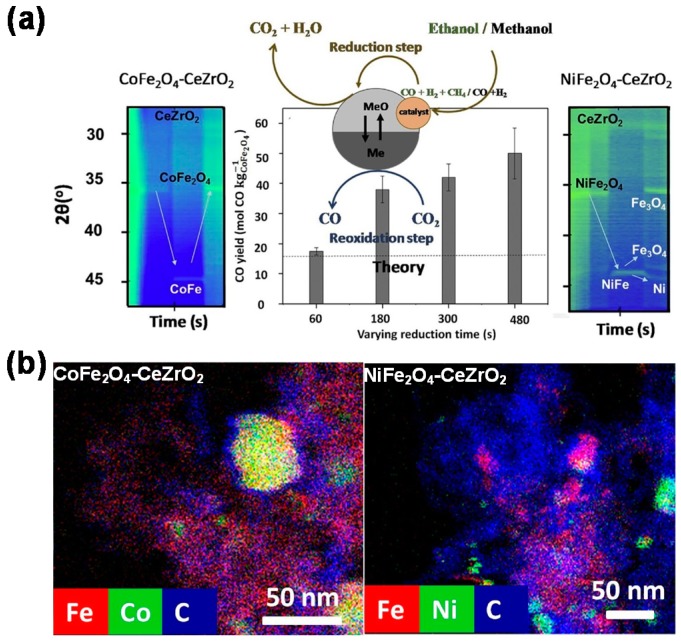
Catalyst-assisted chemical looping dry reforming with alcohol fuels as feed over Co- and Ni-ferrites modified with CeZrO_2_ bifunctional materials: (**a**) Overview of the transformation behavior of gas and solid phases; (**b**) STEM-EDX mappings of Co, Ni, Fe and C in 20 wt % CoFe_2_O_4_- or NiFe_2_O_4_-CeZrO_2_ after methanol redox cycles. Reproduced with permission from Dharanipragada N.V.R.A. et al. [125], Applied Catalysis B: Environmental; published by Elsevier, 2018.

An alternative towards the bifunctional material for CCDR processes is provided by spinel ferrites (AFe_2_O_4_), possessing both a catalytic element (A) and an oxygen carrier element (Fe). This ferrite-based material is readily prepared via co-precipitation of all elements, unlike the additional modification of a Fe-based oxygen carrier by a catalyst component. Various Me-ferrites (Me = Ni, Co, Cu, Mn, Zn) have been investigated in chemical looping processes [112,150,151], among which the NiFe_2_O_4_ and CoFe_2_O_4_ show certain catalytic activity and redox stability during chemical looping dry reforming of CH_4_. Given the increase of alcohol feedstocks from chemical conversion of biomass, these ferrite-based bifunctional materials were further tested in chemical looping reforming of alcohol fuels, such as methanol and ethanol, for the production of value added fuels such as syngas. Dharanipragada et al. [125] investigated the performance of NiFe_2_O_4_- or CoFe_2_O_4_-CeZrO_2_ bifunctional materials in a CCDR process with two different alcohol fuels, methanol and an ethanol-water mixture (1:1 molar ratio). In this case (Figure 13), methanol or ethanol acts as feed in the reduction half-cycle, which is first converted into CO and H_2_ (as well as some CH_4_ when using the ethanol-water mixture), that simultaneously reduce the material, yielding CO_2_ and H_2_O. In the re-oxidation half-cycle, CO_2_ is fed to regenerate the material and produce CO.

During prolonged cycling, the material suffers from deactivation due to phase segregation and particle sintering. It was found that CoFe_2_O_4_-CeZrO_2_ with low ferrite content (20 wt %) had good redox activity and stability, but the material with 80 wt % CoFe_2_O_4_ underwent deactivation owing to phase segregation into Co and Fe_3_O_4_. On the other hand, all NiFe_2_O_4_-CeZrO_2_ materials suffered from phase segregation as well as sintering, and the separated Ni and Fe_3_O_4_ phases could not be restored into the original spinel phase (NiFe_2_O_4_). In the methanol-based CCDR process, carbon formation was observed on both NiFe_2_O_4_- and CoFe_2_O_4_-CeZrO_2_, but significantly more intense on the former (Figure 13b), indicating a lower carbon-resistance ability of the Ni-ferrite. The deposited carbon in turn enhanced the CO yield during the re-oxidation half-cycle, because of the reaction between CO_2_ and carbon. This phenomenon was verified experimentally, showing a more elevated CO yield than the theoretical value after material re-oxidation (as seen in Figure 13a). Furthermore, it was suggested that air or O_2_ would be needed for complete gasification of the deposited carbon.

**Figure 14:**
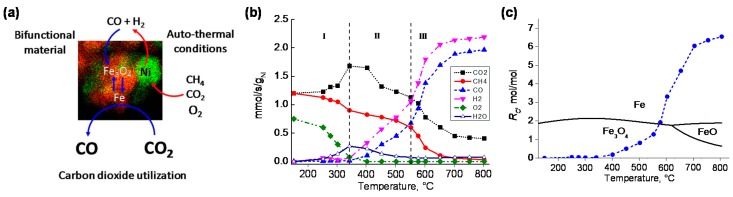
Catalyst-assisted chemical looping auto-thermal dry reforming over a 9 wt % NiO-16 wt % Fe_2_O_3_/MgAl_2_O_4_ bifunctional material: (**a**) Schematic representation of the reactions in the two half-cycles; (**b**) Space-time yield of reagents and products as a function of temperature during reforming of a mixture with molar ratio CH_4_:CO_2_:O_2_ = 1:1:0.5 over the bifunctional material. Region I: total oxidation, region II: dry and steam reforming, region III: auto-thermal dry reforming; (**c**) The ratio *R_c_* between reducing gases (CO + H_2_) and oxidizing gases (CO_2_ + H_2_O) as a function of reaction temperature during catalyst-assisted chemical looping auto-thermal dry reforming process, based on the experimental data of (b). Solid circles with dashed line as guide to the eye. Solid lines: Equilibrium lines of the iron/iron oxide system for each *R_c_* at different temperatures.

Of course, the deactivation of such bifunctional materials due to carbon deposition during CCDR cannot be ignored. In order to tackle this drawback, the upgraded technology of CCAR can provide a prospective solution. The feasibility of the CCAR process over a Ni-Fe/MgAl_2_O_4_ bifunctional material was studied through both thermodynamic analysis and prolonged cycling experiments [20]. In the reduction half-cycle, a mixture of CH_4_, CO_2_ and O_2_ in a 1:1:0.5 molar ratio was first converted over the Ni catalyst into a mixture of CO and H_2_, which both reduce iron oxide to metallic Fe (Figure 14a). The high endothermicity of dry reforming as well as the coke formation could be mitigated in this half-cycle under auto-thermal conditions. The steady-state reforming of the CH_4_:CO_2_:O_2_ =1:1:0.5 mixture over Ni-Fe/MgAl_2_O_4_ at various temperatures showed the auto-thermal dry reforming reduction regime was established in the temperature range from 550 °C to 750 °C, where the O_2_ is completely consumed and the conversion of CH_4_ and CO_2_ respectively reaches 90% and 80%, yielding syngas with a H_2_:CO ratio close to unity (Figure 14b). During the re-oxidation half-cycle, CO is produced alongside the regeneration of iron oxide by CO_2_ (Figure 14a). The overall CO yield depends on the degree of Fe_3_O_4_ reduction reached in the reduction half-cycle, which strongly depends on the ratio (*R_c_*) between the present reducing (CO + H_2_) and oxidizing (CO_2_ + H_2_O) gases. Based on thermodynamic analysis, high conversion of Fe_3_O_4_ to metallic Fe can be reached if *R_c_* > 2 and T > 600 °C in the present study (Figure 14c).

**Figure 15:**
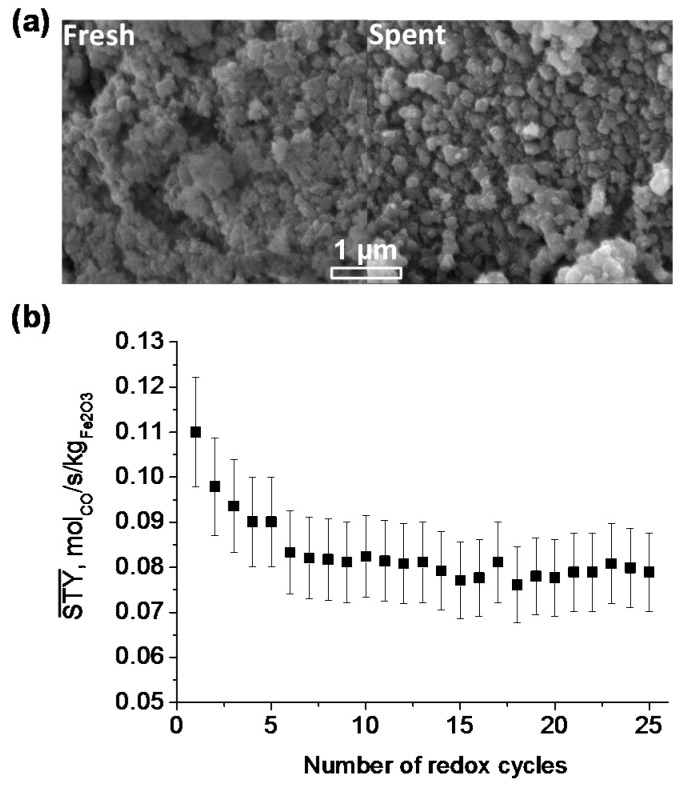
Stability of catalyst-assisted chemical looping auto-thermal dry reforming over the Ni-Fe/MgAl_2_O_4_ bifunctional material: (**a**) SEM micrographs of the fresh sample and the spend sample after 25 cycles; (**b**) Comparison of CO space-time yield ($\bar{\mathrm{STY}}$) during 25 cycles at 750 °C.

The stability of the Ni-Fe/MgAl_2_O_4_ bifunctional material was determined by examining the changes in morphological structure by SEM (Figure 15a), showing no obvious carbon deposition on the material surface, but an increase in the particle size and a collapse in the pore structure after 25 cycles of CCAR. Therefore, particle sintering results in a decrease of the CO time-averaged space-time yield, which reaches a stable level after the first 5 cycles (Figure 15b), suggesting that severe sintering of particles only occurs in the initial stage of redox cycling.

**Figure 16:**
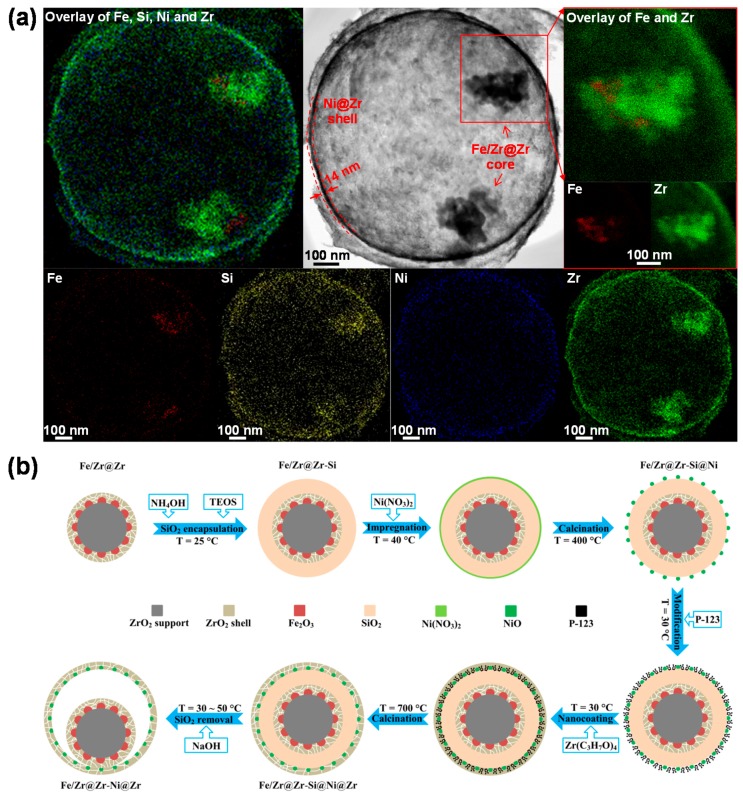
Core-shell bifunctional nanomaterial Fe/Zr@Zr-Ni@Zr: (**a**) STEM micrograph and EDX element mapping of Zr, Si, Ni and Fe, and the overlay mapping of these elements for the as-prepared material; (**b**) Schematics of the synthesis procedure, combining methods of incipient wetness impregnation, SiO_2_ template-assistance and nanocoating.

Obviously, deactivation of bifunctional materials through particle sintering during chemical looping remains a challenge. As mentioned in Section 3.3.2, a possible countering strategy could be the construction of material with a core-shell structure. Hence, a Ni-Fe bifunctional nanomaterial (Fe/Zr@Zr-Ni@Zr) was designed, specifically for the catalyst-assisted chemical looping process. It consists of a ZrO_2_-coated Ni outer shell, encapsulating a Fe_2_O_3_/ZrO_2_@ZrO_2_ core (Figure 16a), showing multiple benefits: (1) the Ni-based shell and the Fe-based core serve as reforming catalyst and oxygen carrier, respectively, and achieve catalytic reforming and oxygen transfer in a single nanoscale unit; (2) the hollow sphere surrounding the core prevents the aggregation of core particles [152], while providing space for gas-solid contact; (3) at the same time, the sphere offers a large specific surface area, leading to fine dispersion of Ni particles and the adequate deposition of the outer ZrO_2_ protective layer, both of which contribute to a high catalytic activity [153]. To design such a structure, an extra step is introduced in the synthesis pathway, where SiO_2_ is typically used as a template [154]: after synthesis of the Fe_2_O_3_/ZrO_2_@ZrO_2_ core, the latter is coated with a dense SiO_2_ layer using a modified Stöber process [155]. After loading the Ni@ZrO_2_ shell, the SiO_2_ template is selectively removed by dissolution with an adequate solvent to free the core, so that it can contact with reactant gases. The result is an eccentric core-shell structure as the core is now no longer supported within the hollow shell (Figure 16b). 

**Figure 17:**
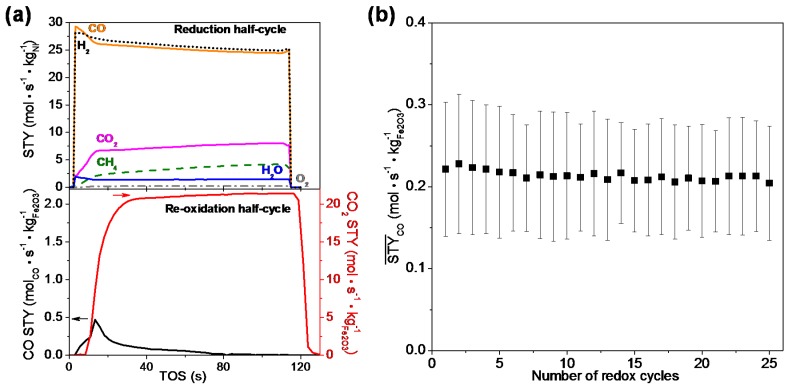
Reactivity and stability of the Fe/Zr@Zr-Ni@Zr bifunctional material during the catalyst-assisted chemical looping auto-thermal dry reforming process at 750 °C: (**a**) Space-time yield (STY) of products vs. time on stream (TOS) during 2 min reduction half-cycle (reactant: mixture of CH_4_:CO_2_:O_2_ = 1:1:0.2, 150 mL⋅min^−1^) and 2 min re-oxidation half-cycle (reactant: 90% CO_2_/Ar, 150 mL⋅min^−1^); (**b**) CO time-averaged space-time yield ($\bar{\mathrm{STY}}$) of the re-oxidation half-cycle during 25 redox cycles.

The bifunctional material Fe/Zr@Zr-Ni@Zr, made according to this recipe, is active for reforming of a feed mixture of CH_4_, CO_2_ and O_2_ (molar ratio of 1:1:0.2) as well as for conversion of CO_2_ to CO in a CCAR process (Figure 17a). O_2_ conversion during reduction is always 100% in these conditions. Methane dry reforming is the dominant reaction, yielding syngas with a H_2_:CO ratio of unity, together with some methane combustion into CO_2_ and H_2_O. In the second half-cycle, the re-oxidation of Fe into Fe_3_O_4_ rapidly converts CO_2_ to CO. Hence, the CO space-time yield shows a sharp peak in the first 20 s of the re-oxidation half-cycle and then steadily decreases towards zero, indicating the majority of Fe_3_O_4_ has been regenerated. Moreover, when averaged over 25 CCAR cycles, a stable CO time-averaged space-time yield is obtained over Fe/Zr@Zr-Ni@Zr (Figure 17b). Characterizations (XPS, STEM-EDX mapping and XRD) of the spent sample after 25 cycles indicate no obvious carbon deposits on the core-shell bifunctional material and no sintering of the Ni and Fe particles.

The CO_2_ utilization during this process can be further enhanced through the construction of a double-zone reactor bed configuration [126], composed of a core-shell structured Fe/Zr@Zr-Ni@Zr bifunctional material (the first zone) followed by a core-shell structured Fe_2_O_3_/ZrO_2_@ZrO_2_ oxygen carrier (the second zone). In this bed configuration, it is expected that much more CO_2_ can be converted to CO due to the additional oxygen storage capacity provided by the second oxygen carrier zone.

## 5. Conclusions

Chemical looping technology has recently emerged as a promising “clean energy conversion technology” due to the advantages of manageable inherent CO_2_ capture. In a chemical looping scheme, a single reaction is broken down into two, spatially or temporally separated half-cycle reactions, coupled through the cyclic transformation of a solid looping material. Depending on their functions, the looping materials can be divided into two main types, correspondingly defining two groups of chemical looping processes: As an oxygen carrier, to transport oxygen through cyclic reduction and re-oxidation during chemical looping combustion or reforming; as a CO_2_ carrier, to remove CO_2_ in-situ through cyclic carbonation and calcination during sorption-enhanced chemical looping. The flexibility of chemical looping processes allows the utilization of various feedstocks (fossil fuels, CO_2_ and renewables) and the production of various value-added chemicals (H_2_, CO, syngas and liquid fuels) via tailoring of reactor feed and looping materials.

At the heart of chemical looping technology is the selection of looping materials. The employment of a suitable looping material determines the sustainability and economics of the technology. From a practical point of view, some commonly used looping materials were compared. CaO is an ideal material to serve as CO_2_ carrier owing to its low cost, non-toxicity, acceptable CO_2_ capacity, regenerability and heat conduction. Further efforts for a CaO-based carrier should focus on increasing its CO_2_ capacity and adsorption rate, as well as improving its thermal stability and mechanical strength. Transition metals, such as Ni, Fe, Cu, Mn and Co-based materials, are commonly used as oxygen carriers, among which Fe- and Ni-based materials are most employed, especially for carbonaceous feedstock-driven chemical looping processes. Iron oxide is highly tolerant to carbon deposition, environmentally friendly, cheap, and has a high oxygen storage capacity from CO_2_. A future task in the development of Fe-based oxygen carriers is the enhancement of its ability to resist sintering. Ni then again has excellent reactivity towards hydrocarbon fuels, making Ni-based material a promising redox catalyst for chemical looping reforming, but the drawbacks with regard to particle sintering, carbon deposition and sulfur poisoning of the material need to be further addressed.

Based on different purposes of improvement of oxygen carriers, approaches have been investigated, mainly focusing on compositional and structural optimization. The stabilization of active components with promoter materials is a widely used method. Some physical promoters (e.g., Al_2_O_3_) can react with the active component to form spinel phases (such as NiAl_2_O_4_ and FeAl_2_O_4_), which can continue to act as inert binders but resulting in the partial loss of reactivity. Adding an appropriate amount of MgO into NiO/Al_2_O_3_ or Fe_2_O_3_/Al_2_O_3_ systems can mitigate the formation of Ni- or Fe-Al_2_O_4_ spinel through the competition of MgAl_2_O_4_ formation. Owing to the thermal and chemical stability, both MgAl_2_O_4_ and ZrO_2_ are considered as a good physical promoter materials for oxygen carriers. In addition, reducible CeO_2_ can serve not only as a physical, but also a chemically active promoter. The CeO_2_-modified oxygen carriers show high reactivity, good reducibility, high oxygen transport capacity as well as strong carbon resistance.

Another strategy is the use of an oxygen carrier with mixed active metals, combining the complementary advantages of individual metals. The mixing of Co into a Ni-based oxygen carrier can improve its reducibility and carbon resistance. The addition of Fe into a Cu-based oxygen carrier increases its melting point to resist agglomeration. Combining advantages of both Ni and iron oxide, the bimetallic Ni/Fe-based material—containing an optimized molar ratio of Ni/Fe and supported by a stable promoter material—gradually becomes a highly promising oxygen carrier or redox catalyst for chemical looping reforming, showing high reactivity towards hydrocarbon fuels, good thermal stability, high ability to resist carbon deposition and low cost.

Structural optimization is of equal importance for an ideal oxygen carrier. Perovskite-structured oxide (ABO_3_-type) materials show remarkable results. Owing to the unique structure of perovskite, its lattice oxygen is readily utilized for syngas production with high selectivity, and phase transformation can be avoided due to its thermal stability. The partial substitution of A- and B-site metals by an aliovalent metal can improve the stability and reactivity, respectively. One optimum composition of perovskite-structured oxygen carriers is La_0.7_Sr_0.3_Cr_0.1_Fe_0.9_O_3_. Future work must focus on improvement of the oxygen storage and transport capacities of perovskite-structured oxygen carriers. Furthermore, a core-shell structure, i.e., an active core encapsulated by a thin and porous shell, has become the most competitive structure, due to its strong adjustability: Control of the particle size of the active core enables to control the reaction kinetics; Tailoring of the composition, thickness and pore properties of the shell can change the stability of the material and its selectivity towards specific products. An ideal core-shell structured oxygen carrier applied in chemical looping should show high oxygen transport capacity and strong thermal stability under the harsh operation conditions. In this sense, a nano-size active metal core encapsulated by a chemically inert and porous shell is required to construct a core-shell structured oxygen carrier. A proposed Fe_2_O_3_/ZrO_2_@ZrO_2_ oxygen carrier, composed of a stable Fe_2_O_3_/ZrO_2_ core and a thin and mesoporous ZrO_2_ shell, proved to possess excellent redox activity and stability for CO_2_ utilization during a long-term chemical looping process.

Efficient conversion of hydrocarbon fuels to syngas accompanied by a goal of maximum CO_2_ utilization has been achieved through a novel process—catalyst-assisted chemical looping dry or auto-thermal dry reforming—implemented over a bifunctional material. The latter must show functions of both reforming catalyst and oxygen carrier. The performances of series of Ni-Fe bifunctional materials were investigated in terms of syngas production, CO_2_ conversion and material stability. A core-shell structured Fe/Zr@Zr-Ni@Zr bifunctional material, providing catalyst and oxygen storage functions from a Ni-based shell and a Fe-based core, respectively, shows a stable CO yield during prolonged cycling without significant carbon deposition and particle sintering. On the reactor scale, careful distribution of the Ni-based catalyst and Fe-based oxygen carrier in the reactor bed could further enhance CO_2_ utilization.

## 6. Outlook

Although chemical looping materials have now been developed as core-shell structured bifunctional nanomaterial, applicable in the new process of chemical looping technology—catalyst-assisted chemical looping, the research on the industrially-applicable looping materials has not yet reached its final destination. Further outlooks could be as follows:

(1) Considering the economic effectiveness, the establishment of low-cost and facile synthesis methods is crucial for the development of the industrial looping materials, especially for the core-shell structured nanomaterials.

(2) High-pressure operation of the chemical looping reactors is essential to maintain the circulation efficiency of an industrial platform. Hence, holistic evaluation of the effect of pressure on the behavior of the core-shell structured materials is necessary for further improvement of the materials.

(3) Research towards the enhancement of the long-term stability, for example maintaining reactivity and structural integrity for thousands of redox cycles, of the looping materials is crucial. The choice of the developed bifunctional looping materials should be screened by taking into account of their lifecycle and cost of production.

(4) Most of the fuel feedstocks contain sulfur-based contaminates, which easily bind to the active metal sites to form stable surface oxy-sulfides and/or bulk metal sulfides, resulting in the material deactivation. Therefore, study on the sulfur-poisoning mechanism of the materials by using feed compositions that are close to real feedstocks is indispensable.

## Figures and Tables

**Table 1:** Summary of the improvement approaches for the different types of oxygen carriers.

Oxygen Carrier(Active Metal)	Promotors/Support	Preparation	Structure	Process	Reactant Gas		Ref.
					Reducing	Oxidizing	
Monometallic-Based Material							
NiO	α-Al_2_O_3_	incipient wetness impregnation	supported	CLC	CH_4_ or 30%CH_4_/N_2_	air or 5%O_2_/N_2_	[64,65]
	La-Co/γ-Al_2_O_3_	impregnation	supported	CLC	CH_4_	air	[66]
	Al_2_O_3_, sepiolite, SiO_2_, TiO_2_, ZrO_2_	mechanical mixing	mixed	CLC	70%CH_4_/H_2_O	air	[58]
	SiO_2_, MgAl_2_O_4_	dry impregnation	supported	CLC and CLR	10%CH_4_ 10%H_2_O 5%CO_2_ 75%N_2_	5%O_2_/N_2_	[59]
	TiO_2_	incipient wetness impregnation	supported	CLC	20%CH_4_/N_2_	air	[67]
	ZrO_2_, TiO_2_, SiO_2_, Al_2_O_3_, NiAl_2_O_4_	wet impregnation	supported	CLR	CH_4_/H_2_O (1/3)	air	[68]
	ZrO_2_, γ-Al_2_O_3_	wet impregnation	supported	CLR	CH_4_/H_2_O (1/3)	air	[69]
	Mg-stabilized ZrO_2_	freeze granulation	supported	CLC CLR	CH_4_/N_2_ CH_4_/H_2_O	5%O_2_/N_2_	[70]
	Yttria-stabilized ZrO_2_	sol-gel and dissolution	mixed	CLC	H_2_	air	[71]
	NiAl_2_O_4_	sol-gel	spinel	CLC	17%CH_4_/He	air	[72]
	NiAl_2_O_4_	spray-drying	mixed	CLC	50%CH_4_/H_2_O	5%O_2_/N_2_	[73]
	MgAl_2_O_4_	freeze granulation	supported	CLR	Nature gas	air	[74]
	α-Al_2_O_3_, MgAl_2_O_4_, CaAl_2_O_4_	dry impregnation	supported	CLC	10%CH_4_ 20%H_2_O 70%N_2_	air	[75]
	CaAl_2_O_4_	impregnation	supported	CLC	15%CH_4_/N_2_	10%O_2_/N_2_	[76]
	CeO_2_, La_2_O_3_	incipient wetness impregnation	supported	CLC	17%CH_4_/Ar	air	[77]
Fe_2_O_3_	γ-Al_2_O_3_	impregnation	supported	CLC	CH_4_	air	[78]
	α-Al_2_O_3_	sol-gel combustion	mixed	CLC	H_2_	air	[79]
	Al_2_O_3_	sol-gel	mixed	CLC	lignite	air	[80]
	γ-Al_2_O_3_	impregnation	supported	CLC	H_2_S ppm/CH_4_	air	[81]
	Al_2_O_3_	co-precipitation	mixed	CL *^a^*	H_2_	CO_2_	[82]
	MgO	theoretical study	supported	CLC	CO	O_2_	[83]
	TiO_2_	incipient wetness impregnation	supported	CLC	CH_4_	O_2_	[84]
	TiO_2_	solid-state mixing	mixed	CLC	3%H_2_/Ar	air	[85]
	TiO_2_	mechanical mixing	mixed	CLC	syngas	H_2_O	[86]
	SiO_2_	dry impregnation	supported	CLR	50%CH_4_/H_2_O	5%O_2_/N_2_	[87]
	MgAl_2_O_4_	freeze granulation	supported	CLC	50%CH_4_/H_2_O	air	[88]
	MgFeAlO_x_	co-precipitation	spinel	CL	5%H_2_/He	CO_2_	[89]
	MgAl_2_O_4_	sequential wetness impregnation	supported	CL *^a^*	CO	H_2_O	[90]
	MgAl_2_O_4_	spray-drying	mixed	CLR	CH_4_	H_2_O	[91]
	CaO	wet impregnation	doped	CLC	10%CO/N_2_	20%CO_2_/N_2_	[92]
	CaO	mechanical mixing	mixed	CLC	10%CO/N_2_	20%CO_2_/N_2_	[93]
	ZrO_2_	co-precipitation	mixed	CLC	10%CO/N_2_	20%CO_2_/N_2_	[94]
	ZrO_2_	theoretical study	supported	CLC	CO	O_2_	[95]
	ZrO_2_	co-precipitation	mixed	CLC CLR	CO CH_4_	O_2_ H_2_O	[96]
	Ce-, Ca-, Mg-stabilized ZrO_2_	freeze granulation	supported	CLC	CH_4_/CO_2_/CO	O_2_	[54]
	CeO_2_	co-precipitation	mixed	CL *^a^*	5%H_2_/He	CO_2_	[22]
	CeO_2_	co-precipitation	mixed	CLR	CH_4_	H_2_O/N_2_	[97]
	CeO_2_	nanometric colloidal sol technique	Surfacegrafted	CL *^a^*	CO or H_2_/N_2_	O_2_/N_2_	[98]
	CeO_2_	sol-gel	mixed	CLR	CO/H_2_/N_2_	H_2_O/N_2_	[99]
	CeO_2_	co-precipitation	solid solution	CL *^a^*	H_2_	CO_2_	[100]
Mixed oxide-based material							
CoO-NiO	Yttria-stabilized ZrO_2_	dissolution	supported	CLC	CH_4_/H_2_O (1/2)	air	[101]
	Al_2_O_3_	incipient wetness impregnation	supported	CLC	CH_4_	air	[102]
Fe_2_O_3_-CuO	MgAl_2_O_4_	mechanical mixing	mixed	CLC	syngas or nature gas	air	[103]
Fe_x_Mn_(1-x)_O	-	co-precipitation	mixed	CLC	5%CH_4_/He	air	[104]
	-	spray-drying	mixed	CLOU	CH_4_ or syngas	5%O_2_/N_2_	[105]
	Al_2_O_3_	spray-drying	supported	CLOU	CH_4_ or syngas	5%O_2_/N_2_	[106]
Ni_x_Mn_(1−x)_O	-	spray-drying	mixed	CLOU	CH_4_ or syngas	5%O_2_/N_2_	[107]
Fe_2_O_3_-NiO	Al_2_O_3_	sequential impregnation	supported	CLC	25%CH_4_/N_2_	10%O_2_/N_2_	[108]
	CeO_2_, Al_2_O_3_	incipient wetness impregnation	supported	CLC	17%CH_4_/Ar	20%O_2_/He	[109]
	Al_2_O_3_	mechanical mixing and impregnation	supported	CLG *^b^*	biomass	air	[110]
	Al_2_O_3_	co-precipitation	mixed	CLG *^b^*	biomass	air	[111]
	La_0.8_Sr_0.2_FeO_3_	sol-gel	supported	CLR	30%CH_4_/N_2_	30%CO_2_/N_2_	[112]
Perovskite-structured material							
LaFeO_3_	-	Solution combustion	perovskite	CLR	CH_4_/Ar	O_2_/Ar	[113]
La_1__−__x_Sr_x_FeO_3_	-	co-precipitation	perovskite	CLR	CH_4_	H_2_O	[114]
	-	solution combustion	perovskite	CLR	40%CH_4_/N_2_	air	[115]
La_1__−__x_Sr_x_M_y_Fe_1__−__y_O_3_	M = Ni, Co, Cr, Cu	citrate method	perovskite	CLR	CH_4_	O_2_ or H_2_O	[116]
Core-shell structured material							
NiO (core)	SiO_2_ (shell)	SiO_2_ coating	core-shell	DR	CO_2_:CH_4_:N_2_ = 1:1:1		[117]
	Al_2_O_3_ (shell)	atomic layer deposition	core-shell	DR	CO_2_:CH_4_:He = 1:1:8		[118]
Fe_3_O_4_ (core)	SiO_2_ (shell)	microemulsion-based synthesis	core-shell	CLC	50%CH_4_/N_2_	50%O_2_/N_2_	[119]
Fe_2_O_3_ (core)	SiO_2_ (shell)	reverse microemulsion	core-shell	CL *^a^*	H_2_	CO_2_	[120]
	La_1__−__x_Sr_x_FeO_3_ (shell)	sequential sol-gel	core-shell	CLR	CH_4_	O_2_	[121]
MeO_x_ (core) (Me = Mn, Co, Fe)	La_1-x_Sr_x_FeO_3_ (shell)	modified Pechini method	core-shell	CLR	10%CH_4_/He	10%O_2_/He	[122]
LaMn_0.7_Fe_0.3_O_3.15_ (core)	SiO_2_ (shell)	surfactant-templating	core-shell	CLC	CH_4_	air	[123]
CuO (shell)	TiO_2_-Al_2_O_3_ (core)	self-assembly template combustion	core-shell	CLC CLOU	CH_4_ N_2_	air O_2_	[124]
Fe_2_O_3_/ZrO_2_ (core)	ZrO_2_ (shell)	impregnation and nanocoating	core-shell	CL *^a^*	5%H_2_/Ar	CO_2_	[40]
Bifunctional material							
Ni-Fe_2_O_3_	CeO_2_	co-precipitation and impregnation	supported	CCDR	CO_2_:CH_4_ = 1:1	CO_2_	[23]
NiFe_2_O_4_ or CoFe_2_O_4_	CeZrO_2_	co-precipitation	spinel	CCDR	methanol or ethanol-H_2_O	CO_2_	[125]
Ni-Fe_2_O_3_	MgAl_2_O_4_	co-impregnation	supported	CCAR	CO_2_:CH_4_:O_2_= 1:1:0.5	CO_2_	[20]
Ni_(shell)_-Fe_2_O_3(core)_	ZrO_2_	impregnation, nanocoating and SiO_2_-template assisted	core-shell	CCAR	CO_2_:CH_4_:O_2_= 1:1:0.2	CO_2_	[126]

^a^ chemical looping; ^b^ chemical looping gasification.
